# Performance of heart rate adjusted heart rate variability for risk stratification of sudden cardiac death

**DOI:** 10.1186/s12872-023-03184-0

**Published:** 2023-03-22

**Authors:** Su-Peng Yan, Xin Song, Liang Wei, Yu-Shun Gong, Hou-Yuan Hu, Yong-Qin Li

**Affiliations:** 1 Department of Biomedical Engineering and Imaging Medicine, Army Medical University, 30 Gaotanyan Main Street, Chongqing, 400038 China; 2 Department of Cardiology, Southwest Hospital, Army Medical University, Chongqing, 400038 China

**Keywords:** Heart rate, Heart rate variability, Hypertrophic cardiomyopathy, Sudden cardiac death, Risk stratification

## Abstract

**Purpose:**

As a non-invasive tool for the assessment of cardiovascular autonomic function, the predictive value of heart rate variability (HRV) for sudden cardiac death (SCD) risk stratification remains unclear. In this study, we investigated the performance of the individualized heart rate (HR) adjusted HRV (HRV_I_) for SCD risk stratification in subjects with diverse risks.

**Methods:**

A total of 11 commonly used HRV metrics were analyzed in 192 subjects, including 88 healthy controls (low risk group), 82 hypertrophic cardiomyopathy (HCM) patients (medium risk group), and 22 SCD victims (high risk group). The relationship between HRV metrics and HR was examined with long-term and short-term analysis. The performance HRV_I_ was evaluated by area under the receiver operating characteristic curve (AUC) and covariance of variation (CV).

**Results:**

Most of the HRV metrics were exponentially decayed with the increase of HR, while the exponential power coefficients were significantly different among groups. The HRV_I_ metrics discriminated low, medium and high risk subjects with a median AUC of 0.72[0.11], which was considerably higher than that of the traditional long-term (0.63[0.04]) and short-term (0.58[0.05]) HRV without adjustment. The average CV of the HRV_I_ metrics was also significantly lower than traditional short-term HRV metrics (0.09 ± 0.02 vs. 0.24 ± 0.13, *p* < 0.01).

**Conclusions:**

Subjects with diverse risks of SCD had similar exponential decay relationship between HRV metrics and HR, but with different decaying rates. HRV_I_ provides reliable and robust estimation for risk stratification of SCD.

## Introduction

Sudden cardiac death (SCD), defined as “sudden and unexpected death occurring within an hour of the onset of symptoms, or occurring in patients found dead within 24 h of being asymptomatic and presumably due to a cardiac arrhythmia or hemodynamic catastrophe’’, is the most common cause of death worldwide, accounting for 20% of global deaths and 50% of deaths from cardiovascular disease [1]. Despite decades of efforts in public cardiopulmonary resuscitation and quality of emergency medical services, according to the latest data, only about 10.4% victims experienced SCD survived to hospital discharge [2]. These startling figures highlight the significance of early SCD prediction for reducing mortality. Fortunately, clinical studies indicated that these catastrophic events can be predicted and prevented by implementing evidence-based, guideline-endorsed recommendations for primary or secondary prevention of SCD [3].

A major challenge in the prediction/prevention of SCD lies in the ability to identify the minority of patients at high risk and provide reassurance to those deemed to be at low risk [4]. The presence of overt structural, ischemic and/or primary electrical heart disease is associated with major elevations in SCD risk [5]. Additionally, certain individuals are known to be at significantly increased risk for SCD among the general population, including those with family history of SCD, a high-risk mutant gene, hypertrophic cardiomyopathy, reduced left ventricular ejection fraction plus or minus clinical heart failure, known or suspected ventricular arrhythmias, long QT syndrome, brugada syndrome [6]. Despite contemporary risk stratification techniques, prediction/prevention of SCD represent major challenges, because current risk stratification strategies do not provide individualized absolute risk and have a low positive predictive accuracy [7].

Although the causes are varied, different etiologies share ventricular arrhythmias as a final common pathway of SCD. Heart rate variability (HRV) analysis gives information about the state of the autonomic nervous system responsible for regulating cardiac electrical activity [8]. Clinical studies have shown that HRV metrics are the most prominent electrophysiological indicators for SCD risk assessment in patients after cardiac surgery, after myocardial infarction, with ventricular dysfunction, and with arrhythmias [9–12]. Although HRV can be used as an independent prognostic factor in combination with other recognized risk factors in risk stratification, this technique has not been incorporated into clinical practice due to its low reproducibility. The most important reason is that different methodological aspects can affect the quantification, interpretation and comparison of the HRV studies [13]. In particular, HRV metrics is primarily heart rate (HR) dependent and HR significantly influences HRV due to both physiological and mathematical reasons [14, 15]. But previous studies regarding SCD risk assessment did not consider the interaction between HR and HRV [16].

In the present study, we investigated the relationship between HRV metrics and HR in low, medium and high risk subjects, developed an individualized HR adjusted HRV (HRV_I_) approach, and evaluated the reliability and robustness of HRV_I_ for SCD risk stratification.

## Materials and methods

### Ethics approval and consent to participate section

This retrospective study was approved by Ethics Committee of Southwest Hospital of the Army Medical University (approval number: KY2020148), and requirement to obtain informed written consent was waived by Ethics Committee of Southwest Hospital of the Army Medical University (approval number: KY2020148) due to the retrospective properties of the study. The study conformed to the provisions of the Declaration of Helsinki (as revised in 2013).

### Study population

Adult hypertrophic cardiomyopathy (HCM) patients or healthy controls (CON) with Holter ECG recordings greater than 18 h were recruited between May 2017 and November 2019 from department of cardiology of Southwest hospital and served as medium and low risk groups. The diagnostic of HCM is established by imaging, with 2D echocardiography or cardiovascular magnetic resonance (CMR) showing a maximal end-diastolic wall thickness of ≥ 15 mm anywhere in the left ventricle [17]. The CON is defined as whom without history of cardiovascular disease, cerebrovascular disease, neurological disease, respiratory disease, dyslipidemia and diabetes mellitus.

Additionally, victims experienced SCD with ECG recordings or R-wave to R-wave interval (RRI) data greater than 1 h available were obtained from a public (Sudden Cardiac Death Holter Database, SDDB) and a commercial ECG databases (American Heart Association ECG databases, AHADB) and served as high risk group [18, 19].

### Assessment of heart rate variability

The method used for HRV analysis has been described elsewhere and adheres to the standards developed by the Task Force of the European Society of Cardiology and the North American Society of Pacing and Electrophysiology [8]. In brief, the digitized ECG signals were preprocessed to extract the consecutive RRIs by using the algorithm developed for the detection of the R waves (Matlab R2020a, MathWorks Inc., Natick, MA, USA) and verified manually by the investigators. All artifacts and ectopic beats were removed and the resultant missing data were replaced by cubic spline interpolation from the nearest valid data [20].

The following 11 commonly used HRV metrics were calculated according to previously published literatures [21]: (1) Time-domain metrics, include the standard deviation of normally conducted RRIs (SDNN), the root mean square of successive differences in normally conducted RRIs (RMSSD), triangular interpolation of RRI histogram (TINN) and HRV triangular index (HRVTI); (2) frequency‐domain metrics, include total power with frequency < 0.4 Hz (TP), power in low frequency range (0.04–0.15 Hz) (LF), and relative power of the low frequency range in normalized units (nLF); (3) nonlinear dynamic metrics, include Poincaré plot standard deviation perpendicular the line of identity (SD1), ratio of SD1 to Poincaré plot standard deviation along the line of identity (SD2) (SD1/SD2), sample entropy (SampEn) and deceleration capacity of heart rate (DC).

Both long-term and short-term analyses were adopted for these HRV metrics respectively. For long-term HRV analysis (HRV_L_), the metrics were computed from the whole ECG recordings without segmentation. For short-term HRV analysis (HRV_S_), the ECG recordings were decomposed into 5-min segments, the metrics were calculated from each segment respectively, and the average values of all segments were taken as the final HRV_S_ results.

### Relationship between HRV metrics and HR

In order to investigate the relationship between HRV metrics and HR, the mean HR of each segment was calculated. For long-term analysis, the whole ECG recording was regarded as one segment, so each metric corresponded to an average HR in each subject. For short-term analysis, each 5-min segment corresponded to an average HR, and the metrics of segments with similar HR were averaged to obtain the mean value of a specific HR. Specifically, the average HR values and HRV metrics for segments with an average HR between 40–50, 50–60, 60–70, 70–80, 80–90, 90–100, 100–110, 110–120 were calculated. A set of HRV metrics corresponding to the HR of approximately 45, 55, 65, 75, 85, 95, 105, 115 were then obtained for each subject respectively.

### HR-based HRV adjustment with individualized power coefficient

The exponential function was used to quantify the relationship between HRV metrics and HR using the following equation that was proposed by Monfredi et al. [22]:

$${\mathrm{HRV}}_{observed}=\alpha \cdot {e}^{-\beta \cdot {HR}_{\mathrm{m}}}$$

where $\alpha$ is constant, $\beta$ is the fitted exponential power coefficient and *HR*_*m*_ is the mean HR of the segments.

The individualized HR based HRV adjustment (HRV_I_) metrics were calculated based on the fitted exponential power coefficient $\beta$ for each subject, using the following equation:

$${\mathrm{HRV}}_{I}=\frac{HR{V}_{observed}}{{e}^{-\beta \cdot H{R}_{\mathrm{m}}}}\cdot {e}^{-\beta \cdot H{R}_{t}}$$

where *HR*_*t*_ is the target HR for adjustment.

### Statistical analysis

Kolmogorov–Smirnov test was used to check the deviations from normality and homogeneity of variance. Continuous data adhering to normality and homoscedasticity were expressed as the mean ± standard deviation (SD) and analyzed by parametric tests (*t*-test or *z*-test for 2 groups, one-way analysis of variance for 3 groups). Continuous data that did not conform to normality and/or homoscedasticity were expressed as the median (interquartile range [IQR]) and analyzed by the non-parametric tests (Wilcoxon rank-sum test for 2 groups, Kruskal–Wallis rank-sum test for 3 groups). Categorical data were expressed as numbers (proportions, %) and analyzed by χ^2^ test. Multiple pairwise comparisons for continuous variables among the groups were made by post hoc tests (Bonferroni correction). The trend of the HRV metrics with increase of HR was analyzed by performing a nonlinear fit with exponential model, and *R*^2^ of fitting was presented when necessary in figure plots. The reliabilities and robustness of HRV_I_ were evaluated with the area under the receiver operating characteristic curve (AUC) and coefficient of variation (CV). AUCs were compared using the Hanley and McNeil method. Two-sided *p* values 0.05 were considered statistically significant and all analyses were performed with the use of SPSS (version 22; IBM Corp, Armonk, NY, USA).

## Results

A total of 192 cases were included in the study (88 CON, 82 HCM patients and 22 SCD victims (20 from SDDB, 2 from AHADB)) and basic information of the subjects is summarized in Table 1. For subjects in the SCD group, age was unavailable in 6 cases, gender was unavailable in 4 cases and LVWT was unavailable in all cases. LVWT was markedly higher in HCM patients compared to that of CON, but there were no statistical significances in age, gender, artifacts number among groups. Number of premature supraventricular beats in the HCM group was more than in the CON but fewer than in the SCD group. Additionally, number of premature ventricular beats was fewer in HCM and CON groups compared with that of the SCD group.

**Table 1:** Baseline characteristics of the investigated subjects

Variable	CON (*n* = 88)	HCM (*n* = 82)	SCD (*n* = 22)
Age, year	53.00 (36.25,64.75)	52.00 (42.00,61.25)	67.50 (37.00,74.25) 6 NA
Gender, n (%)			
Male	51 (58)	49 (60)	10 (46)
Female	37 (42)	33 (40)	8 (36)
			4 NA (18)
Mean HR, bpm	75.00 (69.17,81.59)	69.64 (62.11,76.65)*	69.64 (55.79,85.52)
ECG duration, h	23.35 (21.23,23.89)	23.64 (22.02,23.73)	7.17 (2.54,17.11) *†
PSVB number, n	12.00 (3.25,43.50)	26.00 (10.50,115.25)*	69.50 (1.00,287.00)
PVB number, n	1.00 (0.00,5.00)	2.50 (1.00,20.50)	526.00 (73.50,1460.75) *†
Artifact number, n	37.50 (1.25,270.25)	5.50 (1.00,63.00)	22.50 (2.00,421.25)
LVWT, mm	10.00 (9.05,10.53)	18.90 (16.99,21.00)*	22 NA

*HCM* Hypertrophic cardiomyopathy, *SCD* Sudden cardiac death, *HR* Heart rate, *ECG* Electrocardiogram, *PSVB* Premature supraventricular beat, *PVB* Premature ventricular beat, *LVWT* Left ventricular wall thickness, *NA* Not available

^*^p < 0.05 compared with CON

^†^p < 0.05 compared with HCM

### HRVL results

**Fig. 1:**
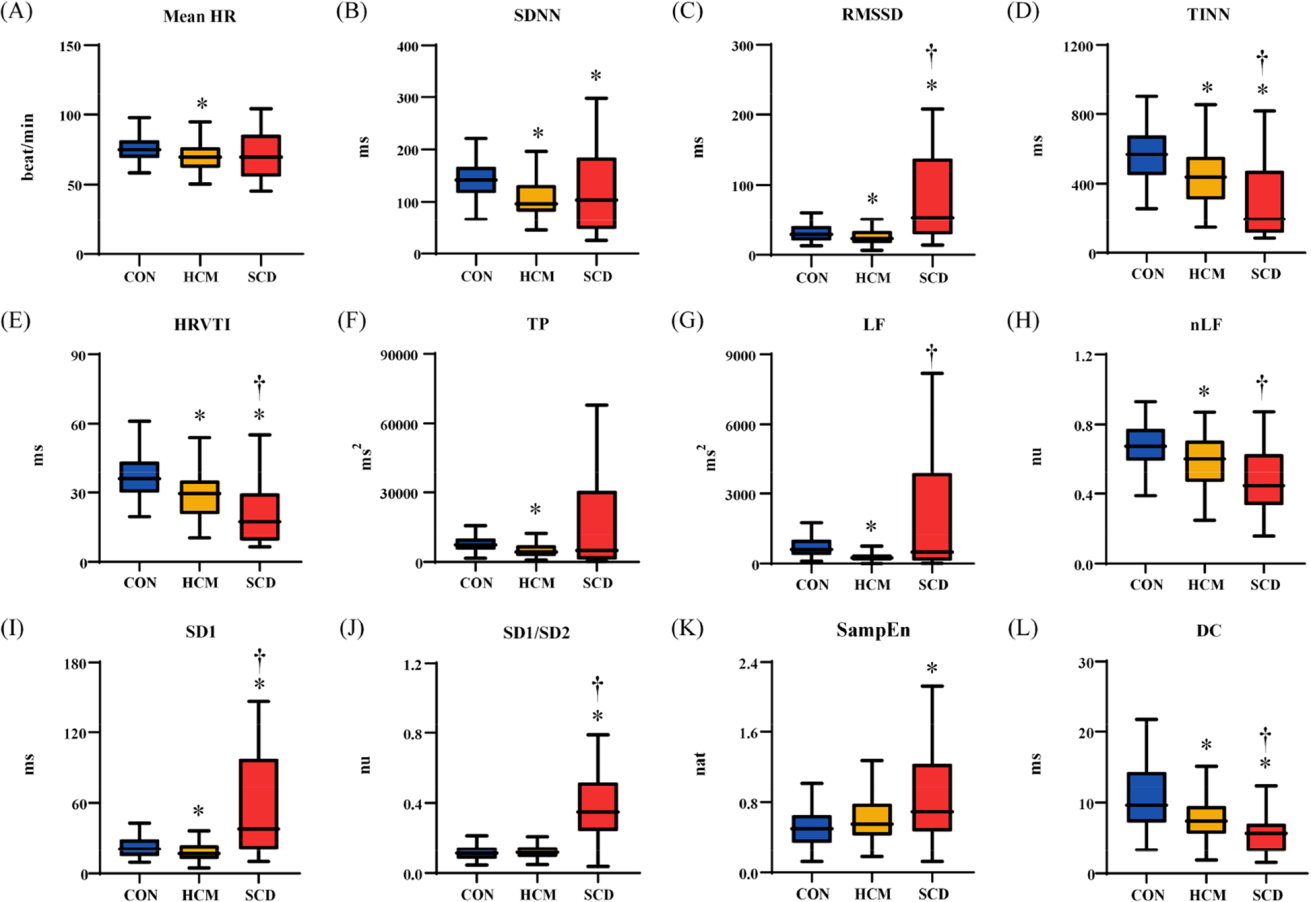
Results of standard long-term heart rate variability analysis. (**A**) mean heart rate (HR); (**B**) standard deviation of normally conducted RR intervals (SDNN); (**C**) root mean square of successive differences in normally conducted RR intervals (RMSSD); (**D**): triangular interpolation of RR intervals histogram (TINN); (E): HRV triangular index (HRVTI); (**F**): total power with frequency < 0.4 Hz (TP); (**G**): power in low frequency range (0.04–0.15 Hz) (LF); (**H**): relative power of the low frequency range in normalized units (nLF); (**I**): Poincaré plot standard deviation perpendicular the line of identity (SD1); (**J**): ratio of SD1 to Poincaré plot standard deviation along the line of identity (SD1/SD2); (**K**): sample entropy (SampEn); (**L**): deceleration capacity of heart rate (DC). CON: healthy control; HCM: hypertrophic cardiomyopathy; SCD: sudden cardiac death. *: *p* < 0.05 compared with CON; †: *p* < 0.05 compared with HCM

The HRV_L_ results are shown in Fig. 1. Mean HR was significantly higher in the HCM than CON group, but did not differ with SCD group. Five of the 11 investigated HRV metrics (RMSSD, TINN, HRVTI, SD1, DC) were differed significantly among the 3 groups. Additionally, 4 metrics (SDNN, TP, LF, nLF) were significantly differed between HCM and CON groups. Two metrics (LF, SD1/SD2) were significantly differed between HCM and SCD groups.

It is worth noting that 3 HRV metrics (TINN, HRVTI, DC) had better performance than others and showed stable decreasing trend among the 3 groups, that is, with a higher value in the CON group, a moderate value in the HCM group and a lower value in the SCD group.

### HRVS results

**Fig. 2:**
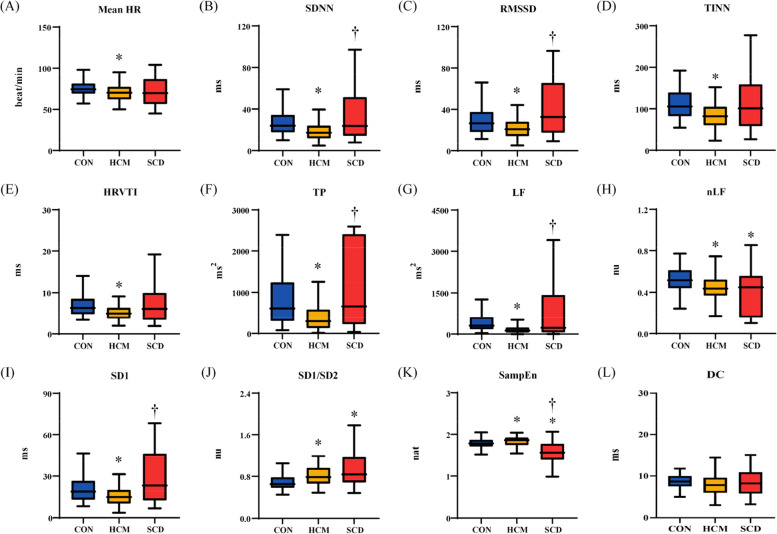
Results of standard short-term heart rate variability analysis. (**A**) mean heart rate (HR); (**B**) standard deviation of normally conducted RR intervals (SDNN); (**C**) root mean square of successive differences in normally conducted RR intervals (RMSSD); (**D**): triangular interpolation of RR intervals histogram (TINN); (E): HRV triangular index (HRVTI); (**F**): total power with frequency < 0.4 Hz (TP); (**G**): power in low frequency range(0.04–0.15 Hz) (LF); (**H**): relative power of the low frequency range in normalized units (nLF); (**I**): Poincaré plot standard deviation perpendicular the line of identity (SD1); (**J**): ratio of SD1 to Poincaré plot standard deviation along the line of identity (SD1/SD2); (**K**): sample entropy (SampEn); (**L**): deceleration capacity of heart rate (DC). CON: healthy control; HCM: hypertrophic cardiomyopathy; SCD: sudden cardiac death.*: *p* < 0.05 compared with CON; †: *p* < 0.05 compared with HCM

The HRV_S_ results are shown in Fig. 2. Mean HR remains significantly higher in the HCM group than CON. Among the 11 investigated HRV metrics, only 1 metric (SampEn) had significant difference among the 3 groups. Nine metrics (SDNN, RMSSD, TINN, HRVTI, TP, LF, nLF, SD1, SD1/SD2) were significantly differed between HCM and CON groups. Five metrics (SDNN, RMSSD, TP, LF, SD1) were significantly differed between HCM and SCD groups.

**Fig. 3:**
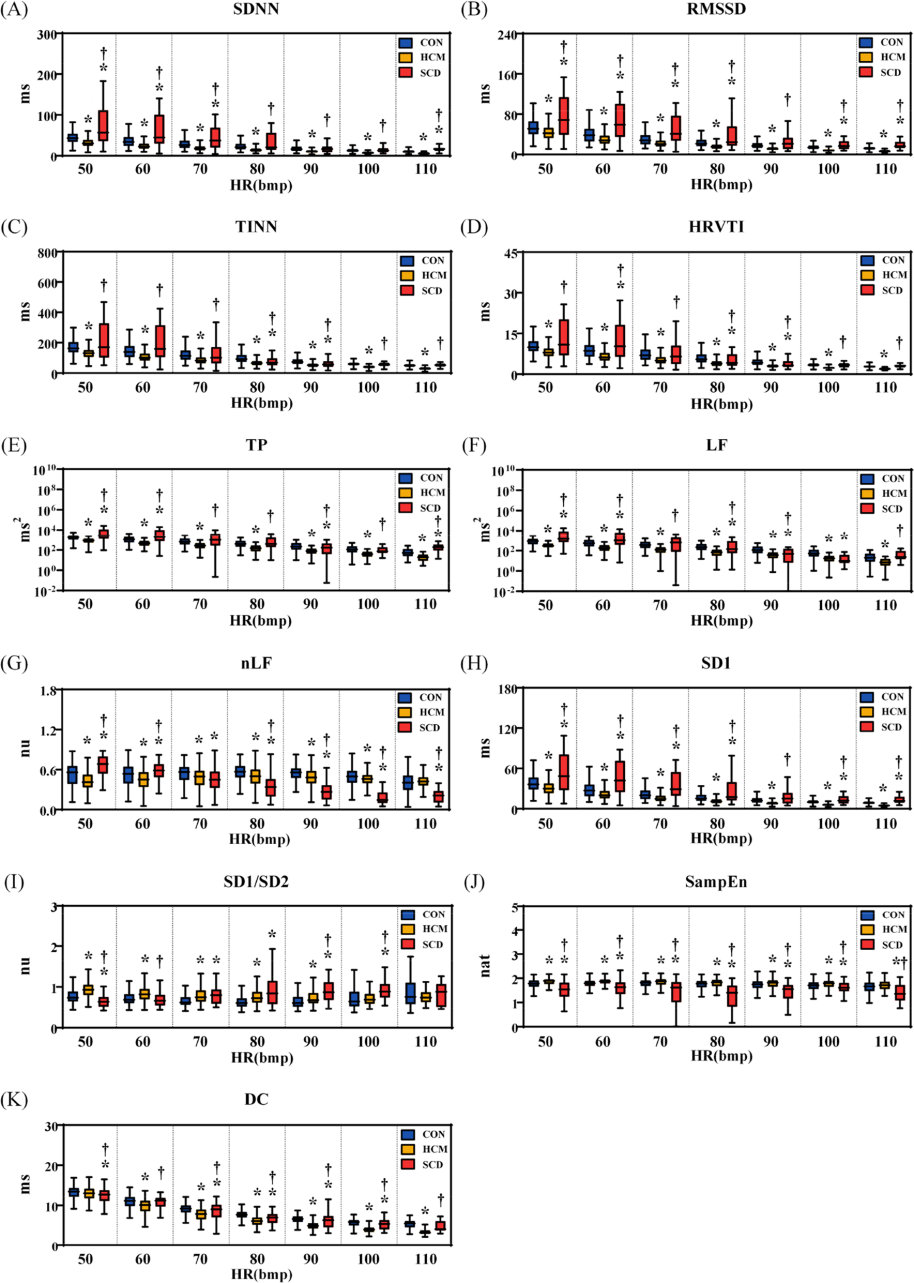
Results of standard short-term heart rate variability analysis at different heart rate (HR) ranges. (**A**) standard deviation of normally conducted RR intervals (SDNN); (**B**) root mean square of successive differences in normally conducted RR intervals (RMSSD); (**C**): triangular interpolation of RR intervals histogram (TINN); (**D**): HRV triangular index (HRVTI); (**E**): total power with frequency < 0.4 Hz (TP); (**F**): power in low frequency range(0.04–0.15 Hz) (LF); (**G**): relative power of the low frequency range in normalized units (nLF); **H**: Poincaré plot standard deviation perpendicular the line of identity (SD1); (**I**): ratio of SD1 to Poincaré plot standard deviation along the line of identity (SD1/SD2); (**J**): sample entropy (SampEn); (**K**): deceleration capacity of heart rate (DC). CON: healthy control; HCM: hypertrophic cardiomyopathy; SCD: sudden cardiac death.*: *p* < 0.05 compared with CON; †: *p* < 0.05 compared with HCM

Figure 3 shows the HRV_S_ results at different HR ranges. The differences of each metric among groups at different HR levels were still significant. Specifically, 6 metrics (SDNN, RMSSD, TINN, HRVTI, TP, LF, SD1) in the HCM group were significantly differed with those of the CON and SCD groups at all of the HR levels. Two metrics (nLF, SampEn) in the SCD group were significantly differed with those of the CON group regardless of HR levels.

### Relationship between HRV metrics and HR

**Fig. 4:**
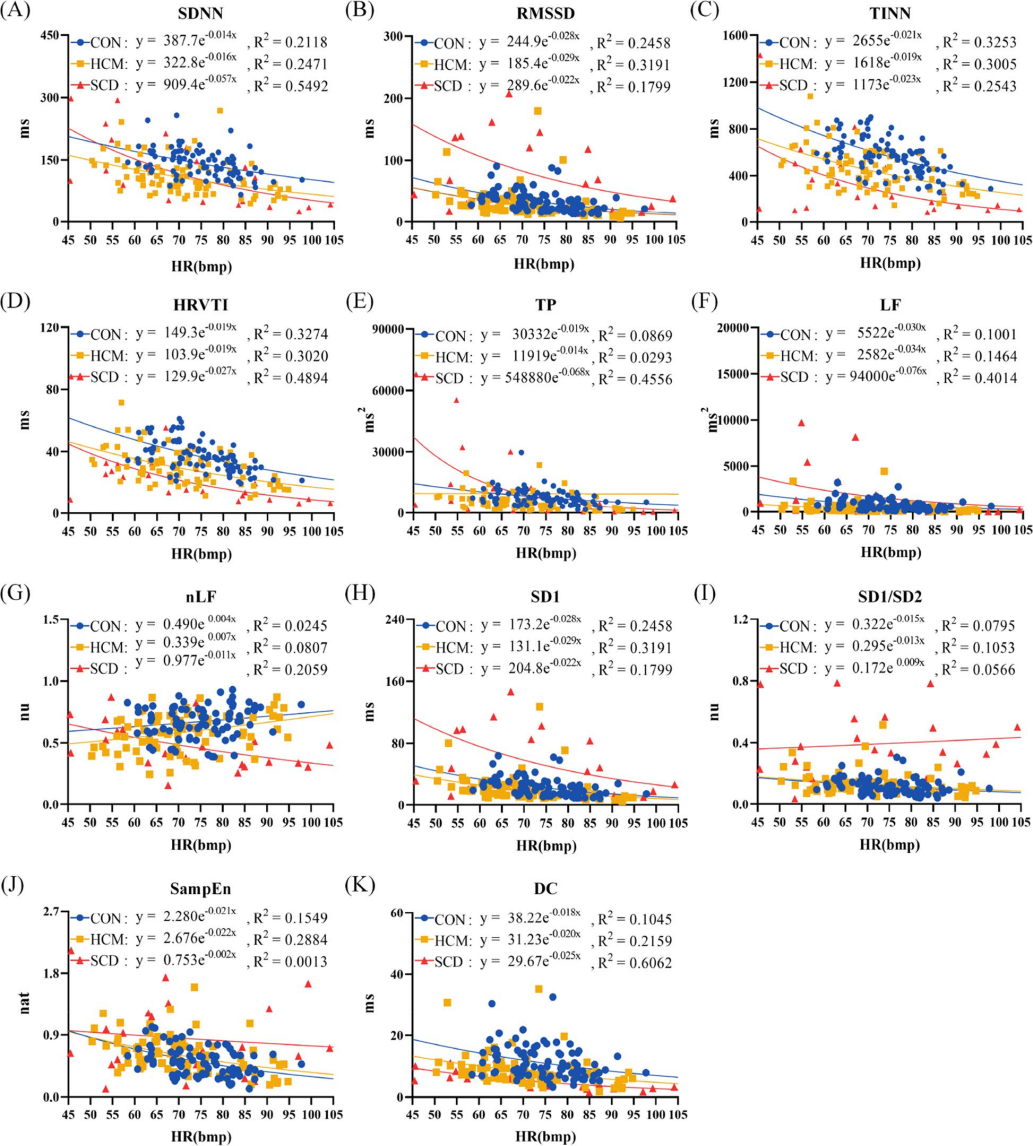
The fitted exponential function curve between long-term heart rate variability analysis metrics and heart rate (HR). (**A**) standard deviation of normally conducted RR intervals (SDNN); (**B**) root mean square of successive differences in normally conducted RR intervals (RMSSD); (**C**): triangular interpolation of RR intervals histogram (TINN); **D**: HRV triangular index (HRVTI); (**E**): total power with frequency < 0.4 Hz (TP); (**F**): power in low frequency range (0.04–0.15 Hz) (LF); (**G**): relative power of the low frequency range in normalized units (nLF); (**H**): Poincaré plot standard deviation perpendicular the line of identity (SD1); (**I**): ratio of SD1 to Poincaré plot standard deviation along the line of identity (SD1/SD2); (**J**): sample entropy (SampEn); (**K**): deceleration capacity of heart rate (DC). CON: healthy control; HCM: hypertrophic cardiomyopathy; SCD: sudden cardiac death

Figure 4 shows the fitted exponential function curve between long-term HRV metrics and HR. An exponential decay-like relationship was observed for all of the investigated metrics in the 3 groups. But the *R*^2^ values were relatively lower for all of the metrics, except that SDNN and DC in the SCD group with a *R*^2^ value greater than 0.5.

**Fig. 5:**
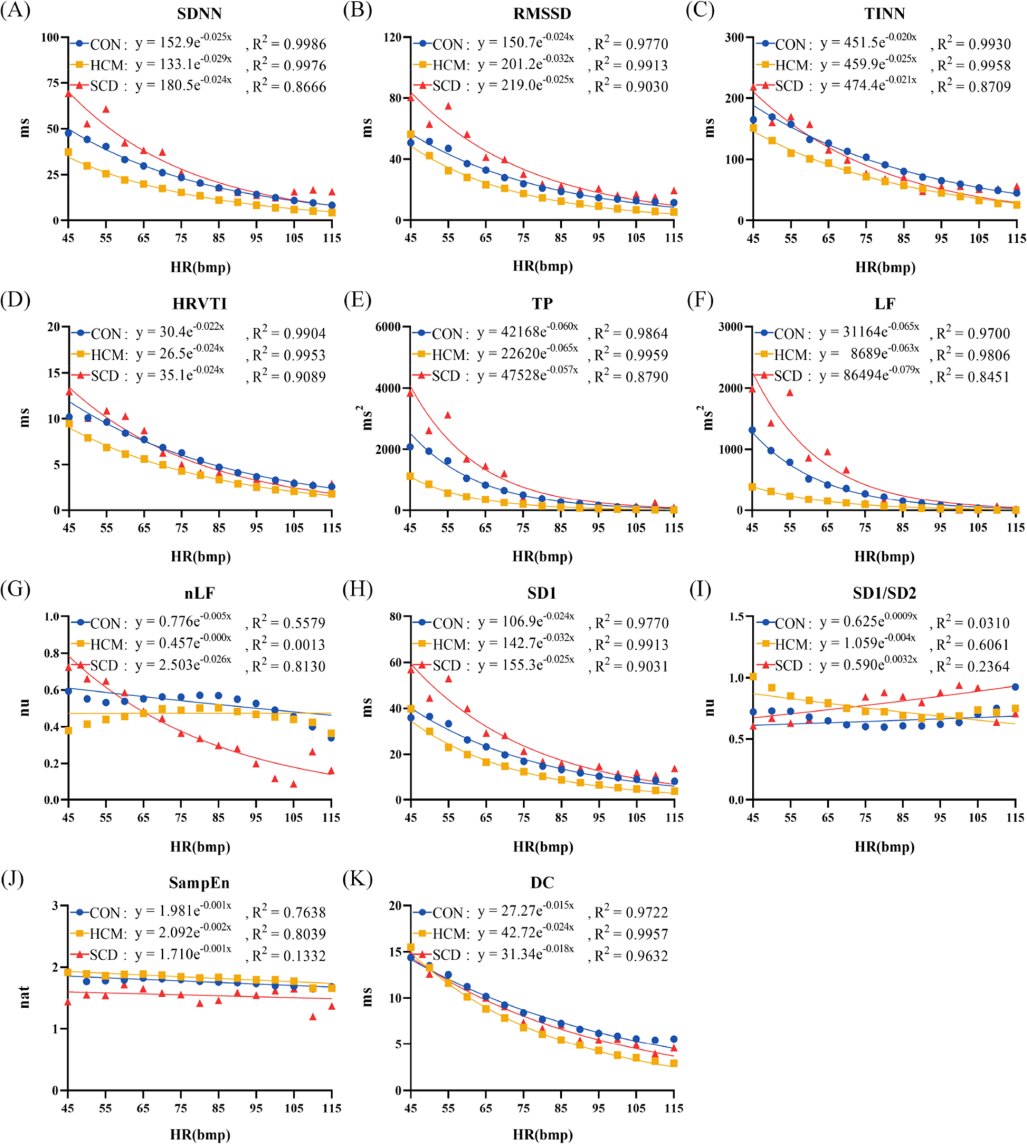
The fitted exponential function curve between short-term heart rate variability analysis metrics and heart rate (HR). (**A**) standard deviation of normally conducted RR intervals (SDNN); (**B**) root mean square of successive differences in normally conducted RR intervals (RMSSD); (**C**): triangular interpolation of RR intervals histogram (TINN); **D**: HRV triangular index (HRVTI); (**E**): total power with frequency < 0.4 Hz (TP); (**F**): power in low frequency range (0.04–0.15 Hz) (LF); (**G**): relative power of the low frequency range in normalized units (nLF); (**H**): Poincaré plot standard deviation perpendicular the line of identity (SD1); (**I**): ratio of SD1 to Poincaré plot standard deviation along the line of identity (SD1/SD2); (**J**): sample entropy (SampEn); (**K**): deceleration capacity of heart rate (DC). ON: healthy control; HCM: hypertrophic cardiomyopathy; SCD: sudden cardiac death

Figure 5 shows the fitted exponential function curve between short-term HRV metrics and HR. Similarly, an exponential decay-like relationship was observed for all of the investigated metrics. The *R*^2^ values of 8 metrics (SDNN, RMSSD, TINN, HRVTI, TP, LF, SD1, DC) were greater than 0.5 in all of the 3 groups. The *R*^2^ values of 3 metrics (SDNN, RMSSD, SD1) were greater than 0.5 in 2 groups and the *R*^2^ value of 1 metric (SD1/SD2) was greater than 0.5 in 1 group.

Table 2 lists the exponential power coefficients that representing the decay rate of short-term HRV metrics with mean HR in each group. One metric (nLF) had significant different coefficients among 3 groups. All metrics except for TP and SampEn had significant different coefficients between HCM and CON groups. Two metrics (SD1/SD2, DC) had significant different coefficients between HCM and SCD groups. Three metrics (RMSSD, TINN, TP) had significant different coefficients between CON and SCD groups.

**Table 2:** Results of individualized power coefficient of exponential function

Variable	CON (*n* = 88)	HCM (*n* = 82)	SCD (*n* = 22)
SDNN, ms	-0.022 (-0.030,-0.016)	-0.028 (-0.036,-0.022)*	-0.029 (-0.060,-0.016)
RMSSD, ms	-0.027 (-0.032,-0.018)	-0.032 (-0.044,-0.025)*	-0.040 (-0.056,-0.021) *
TINN, ms	-0.020 (-0.025,-0.014)	-0.023 (-0.029,-0.019)*	-0.026 (-0.047,-0.019) *
HRVTI, ms	-0.021 (-0.027,-0.015)	-0.026 (-0.031,-0.020)*	-0.026 (-0.041,-0.017)
TP, ms^2^	-0.045 (-0.063,-0.032)	-0.056 (-0.080,-0.041)	-0.080 (-0.122,-0.040) *
LF, ms^2^	-0.037 (-0.058,-0.025)	-0.048 (-0.070,-0.034)*	-0.063 (-0.093,-0.025)
nLF, nu	0.003 (-0.003,0.009)	0.008 (0.002,0.015)*	-0.020 (-0.038,-0.005) * †
SD1, ms	-0.027 (-0.032,-0.018)	-0.032 (-0.044,-0.025)*	-0.040 (-0.056,-0.021) *
SD1/SD2, nu	-0.003 (-0.008,0.004)	-0.005 (-0.013,-0.001)	0.003 (-0.004,0.011) †
SampEn, nat	-0.002 (-0.003,0.001)	-0.001 (-0.005,0.003)	0.001 (-0.010,0.010)
DC, ms	-0.017 (-0.021,-0.014)	-0.025 (-0.029,-0.022)*	-0.019 (-0.031,-0.013) †

*HCM* Hypertrophic cardiomyopathy, *SCD* Sudden cardiac death, *SDNN* Standard deviation of normally conducted RR intervals, *RMSSD* Root mean square of successive differences in normally conducted RR intervals, *TINN* Triangular interpolation of RR intervals histogram, *HRVTI* HRV triangular index, *TP* Total power with frequency < 0.4 Hz, *LF* Power in low frequency range(0.04–0.15 Hz), *nLF* Relative power of the low frequency range in normalized units, *SD1* Poincaré plot standard deviation perpendicular the line of identity, *SD1/SD2* Ratio of SD1 to Poincaré plot standard deviation along the line of identity, *SampEn* Sample entropy, *DC* Deceleration capacity of heart rate, *nu* Normalized unit

^*^p < 0.05 compared with CON

^†^p < 0.05 compared with SCD

### Results of HRVI

**Fig. 6:**
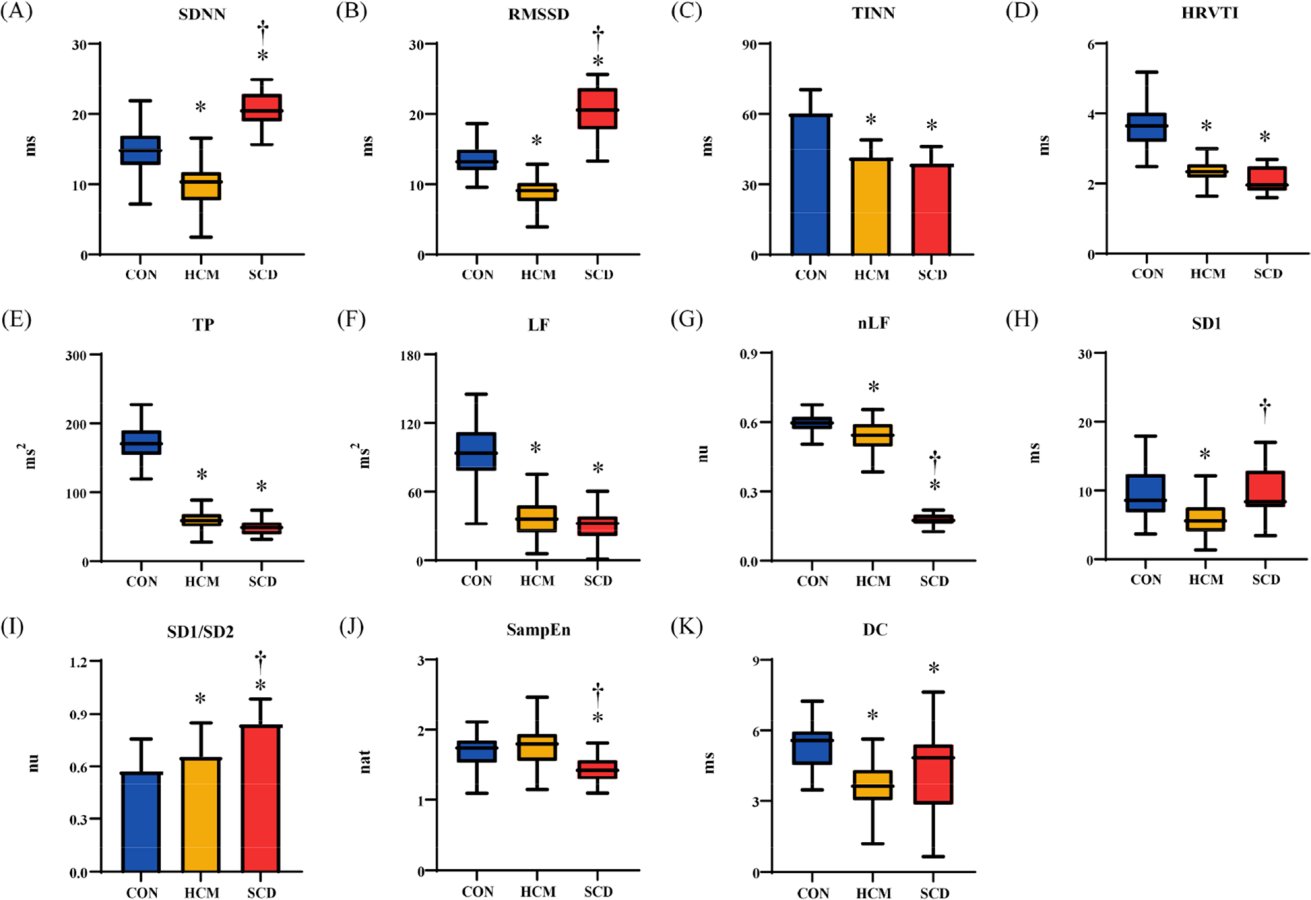
Results individualized heart rate based heart rate variability adjustment metrics. (**A**) standard deviation of normally conducted RR intervals (SDNN); (**B**) root mean square of successive differences in normally conducted RR intervals (RMSSD); (**C**): triangular interpolation of RR intervals histogram (TINN); **D**: HRV triangular index(HRVTI); (**E**): total power with frequency < 0.4 Hz (TP); (**F**): power in low frequency range (0.04–0.15 Hz) (LF); (**G**): relative power of the low frequency range in normalized units (nLF); (**H**): Poincaré plot standard deviation perpendicular the line of identity (SD1); (**I**): ratio of SD1 to Poincaré plot standard deviation along the line of identity (SD1/SD2); (**J**): sample entropy (SampEn); (**K**): deceleration capacity of heart rate (DC). CON: healthy control; HCM: hypertrophic cardiomyopathy; SCD: sudden cardiac death.*: *p* < 0.05 compared with CON; †: *p* < 0.05 compared with HCM

The HRV_I_ results are shown in Fig. 6. After adjustment, 4 metrics (SDNN, RMSSD, nLF and SD1/SD2) had significant differences among the 3 groups, while nLF and SD1/SD2 showed a stable decreasing/increasing trend. Five metrics (TINN, HRVTI, TP, LF, DC) had significant differences between the HCM and CON groups. One metric (SampEn) was significant different between the HCM and SCD groups and 1 metric (SD1) was significant different between the CON and SCD groups.

### Performance of HRVI for SCD risk stratification

Table 3 lists the AUC values of each metric for SCD risk stratification using different HRV analysis methods. There were no significant differences between HRV_L_ and HRV_S_ metrics except that AUCs of SD1/SD2 and DC were relatively lower for HRV_S_. Seven HRV_I_ metrics (SDNN, RMSSD, TINN, HRVTI, TP, LF, nLF) had significantly higher AUC values, either compared to HRV_L_ or compared to HRV_S_.

**Table 3:** AUC values of HRV metric for SCD risk stratification using different HRV analysis methods

Variable	AUC		
	HRV_L_	HRV_S_	HRV_I_
SDNN, ms	0.59 ± 0.03	0.58 ± 0.03	0.84 ± 0.02* †
RMSSD, ms	0.63 ± 0.03	0.59 ± 0.03	0.91 ± 0.02* †
TINN, ms	0.64 ± 0.03	0.58 ± 0.03	0.72 ± 0.03* †
HRVTI, ms	0.64 ± 0.03	0.56 ± 0.03	0.73 ± 0.02*†
TP, ms2	0.52 ± 0.02	0.57 ± 0.03	0.73 ± 0.02* †
LF, ms2	0.60 ± 0.03	0.62 ± 0.03	0.71 ± 0.02* †
nLF, nu	0.63 ± 0.03	0.58 ± 0.03	0.77 ± 0.02*†
SD1, ms	0.63 ± 0.03	0.59 ± 0.03	0.66 ± 0.03
SD1/SD2, nu	0.71 ± 0.02	0.62 ± 0.03*	0.66 ± 0.03
SampEn, nat	0.61 ± 0.03	0.63 ± 0.03	0.66 ± 0.03
DC, ms	0.67 ± 0.03	0.50 ± 0.03*	0.69 ± 0.02†

*AUC* Area under the receiver operating characteristic curve, *HRV* Heart rate variability, *HRV*_*L*_ Standard long-term HRV metrics, *HRV*_*S*_ Standard short-term HRV metrics, *HRV*_*F*_ HRV metrics adjusted by heart rate based on the fixed exponential power coefficient, *HRV*_*I*_ HRV metrics adjusted by heart rate based on the individualized exponential power coefficient, *SDNN* Standard deviation of normally conducted RR intervals, *RMSSD* Root mean square of successive differences in normally conducted RR intervals, *TINN* Triangular interpolation of RR intervals histogram, *HRVTI* HRV triangular index, *TP* Total power with frequency < 0.4 Hz, *LF* Power in low frequency range(0.04–0.15 Hz), *nLF* Relative power of the low frequency range in normalized units, *SD1* Poincaré plot standard deviation perpendicular the line of identity, *SD1/SD2*, Ratio of SD1 to Poincaré plot standard deviation along the line of identity, *SampEn* Sample entropy, *DC* Deceleration capacity of heart rate; nu: normalized unit

^*^p < 0.05 compared with HRV_L_

^†^p < 0.05 compared with HRV_S_

The HRV_I_ metrics discriminated low, medium and high risk subjects with an AUC ranging from 0.66 to 0.91 (median 0.72[0.11]), which were considerably greater than that of HRV_L_ (from 0.52 to 0.71, median 0.63[0.04]), and HRV_S_ (from 0.50 to 0.63, median 0.58[0.05]).

**Fig. 7:**
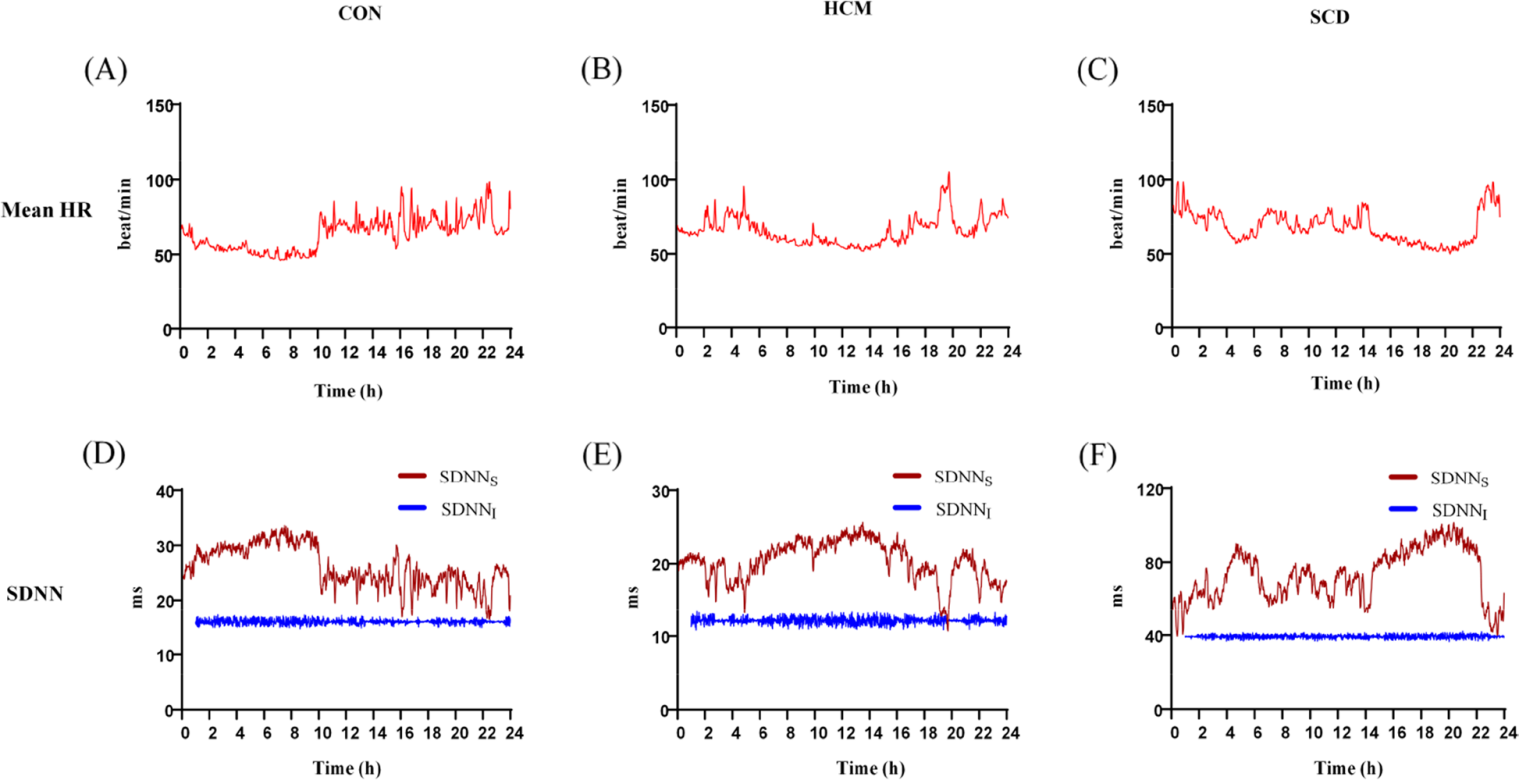
An example of chart of individualized heart rate (HR) based heart rate variability adjustment metric standard deviation of normally conducted RR intervals (SDNN) changes with analysis time. (**A**): mean heart rate (HR) of healthy control (CON); (**B**): mean HR of hypertrophic cardiomyopathy (HCM); (**C**): mean HR of sudden cardiac death (SCD); (D) SDNN calculated from HRV_S_ (SDNN_S_) and HRV_I_ (SDNN_I_) of CON; **E** SDNN_S_ and SDNN_I_ of HCM; **F** SDNN_S_ and SDNN_I_ of SCD

Figure 7 shows an example of the chart of HRVI metric SDNN changes with analysis time. SDNN calculated using traditional HRVS method fluctuated inversely with HR in subjects form each group. After adjustment, SDNN kept constant and was not affected by the analyzing time and duration. Table 4 lists the CV of HRVs and HRVI metrics. All of the HRV_I_ metrics had a significantly reduced CV than HRV_S_ metrics in the 3 investigated groups except that nLF was not differed in HCM and SCD groups. The average CV of HRV_I_ metrics was also significantly lower than that of the HRV_S_ metrics (0.09 ± 0.02 vs. 0.24 ± 0.13, *p* < 0.01).

**Table 4:** Coefficient of variation between HRVs and HRV_I_

Metrics	HRV_S_			HRV_I_		
	CON	HCM	SCD	CON	HCM	SCD
SDNN, ms	0.27 ± 0.14	0.20 ± 0.11	0.19 ± 0.18	0.09 ± 0.03*	0.09 ± 0.03†	0.09 ± 0.02ǂ
RMSSD, ms	0.31 ± 0.14	0.22 ± 0.11	0.20 ± 0.17	0.11 ± 0.03*	0.11 ± 0.03†	0.10 ± 0.03ǂ
TINN, ms	0.25 ± 0.13	0.17 ± 0.09	0.19 ± 0.19	0.12 ± 0.04*	0.12 ± 0.04†	0.09 ± 0.03ǂ
HRVTI, ms	0.24 ± 0.11	0.20 ± 0.09	0.18 ± 0.18	0.11 ± 0.04*	0.10 ± 0.03†	0.09 ± 0.03ǂ
TP, ms^2^	0.59 ± 0.38	0.39 ± 0.21	0.37 ± 0.27	0.11 ± 0.04*	0.10 ± 0.04†	0.11 ± 0.04ǂ
LF, ms^2^	0.51 ± 0.44	0.50 ± 0.89	0.42 ± 0.41	0.10 ± 0.03*	0.09 ± 0.03†	0.10 ± 0.03ǂ
nLF, nu	0.10 ± 0.07	0.21 ± 0.93	0.15 ± 0.13	0.07 ± 0.03*	0.07 ± 0.03	0.09 ± 0.03
SD1, ms	0.30 ± 0.14	0.26 ± 0.13	0.20 ± 0.17	0.11 ± 0.04*	0.10 ± 0.03†	0.11 ± 0.03ǂ
SD1/SD2, nu	0.11 ± 0.07	0.10 ± 0.03	0.14 ± 0.08	0.08 ± 0.03*	0.07 ± 0.05†	0.09 ± 0.03ǂ
SampEn, nat	0.09 ± 0.03	0.10 ± 0.03	0.10 ± 0.04	0.04 ± 0.03*	0.04 ± 0.03†	0.06 ± 0.06ǂ
DC, ms	0.20 ± 0.07	0.17 ± 0.07	0.17 ± 0.14	0.11 ± 0.03*	0.11 ± 0.03†	0.08 ± 0.04ǂ

^*^p < 0.05 compared with HRV_S_ for CON group

^†^*p* < 0.05 compared with HRV_S_ for HCM group

^ǂ^*p* < 0.05 compared with HRV_S_ for SCD group

## Discussion

The present study investigated the reliability and robustness of an individualized HR-based HRV adjustment method for risk stratification of SCD. The main findings of our study are: (1) Most HRV metrics have an exponential decay relationship between HRV and HR, but the decaying rate of each metric is significantly differed among different risk groups; (2) HCM patients show decreased vagal activity and impaired sympatho-vagal balance when the confounding effect of HR is eliminated for HRV analysis; and (3) HRV metrics that adjusted by HR with individualized exponential power coefficient improve the performance of SCD risk classification.

As a non-invasive tool for the assessment of cardiovascular autonomic function and risk of SCD, HRV is widely used for clinical research with either long-term (1–24 h) or short-term (several minutes) analysis. Considered as the “golden standard”, long-term HRV describes the autonomic function change over hours or even longer time spans [23]. Based on retrospective analyses of various risk stratification techniques, SCD was found to be associated with depressed HRV in 24-h recordings and can be predicted by some HRV metrics with relatively high accuracy [24, 25]. For instance, Braunisch et al. [26] observed a U-shaped association between HRVTI and mortality in hemodialysis atrial fibrillation patients. This result might contribute to risk stratification independent of known risk scores in these patients. The most predominant advantage of long-term HRV analysis is its stability, while prominent disadvantages are time consuming, poor timeliness, and methodological difficulties [27, 28]. Short-term HRV analysis can track dynamic changes of cardiac autonomic function within minutes and is a convenient method for the estimation of autonomic status. Many studies have evaluated the value of HRV metrics for SCD prediction with short-term HRV analysis [21, 29, 30]. For example, Hämmerle et al. [31] demonstrated that HRVTI measured in a single 5-min ECG recording in a cohort of patients with atrial fibrillation is an independent predictor of cardiovascular mortality and might be a valuable tool for further risk stratification to guide patient management. The advantage of short-term HRV analysis is that it is easy and convenient to perform under controlled conditions, while the main disadvantage is the poor reproducibility and stability due to the constant fluctuation of cardiovascular autonomic function [27]. Additionally, the predictive power of short-term HRV analysis is lower than that reported for analysis of 24-h recordings and these measures may be used only for screening of high risk patients.

In the past decades, a number of clinical studies have been carried out to evaluate the risk of SCD using both long-term and short-term HRV analysis in patients of medium risks, such as patients with HCM because periodic evaluation of their risk of SCD has been an integral part of clinical management [32–39]. The majority of the studies have reported a reduction in HRV parameters and an impaired parasympathetic autonomic regulation [32, 34, 35, 38, 39]. But other studies showed that the autonomic function was not influenced by HCM and the parasympathetic regulation was preserved in HCM patients compared to that of healthy control subjects [36, 37]. Additionally, the autonomic information assessed by HRV was demonstrated to be not significantly differed between HCM patients with and without cardiovascular events [33]. Since the clinical significance of HRV in patients with HCM remains controversial, further studies are warranted to determine the predictive value of HRV for SCD risk stratification [40].

Two factors may account for the controversies of these studies. The first one is that characteristics of the participants, such as age, gender, medication use, comorbidity, respiration, body positioning, lifestyle, and mental stress, have a confounding effect on HRV metrics [41, 42]. Most notably, as an independent risk factor of mortality for patients with cardiovascular diseases, HR is very sensitive to these characteristics and has been demonstrated to be negatively correlated with HRV metrics [43–45]. The second one is the methodologies of HRV analysis, such as length of the analyzed RRI time series, metrics used to measure HRV, number of enrolled patients and standardization of HRV measurements [13, 15, 46, 47]. Correcting the confounding effects is an effective way to improve the reliability and reproducibility of HRV analysis, especially for HR. Earlier attempts to adjust HRV metrics using linear regression analysis or using coefficient of variation have achieved some success, but the robustness and reproducibility remain unsatisfied [16]. Based on the relationship between HRV metrics and HR, HRV metrics was corrected with an exponential function of fixed power coefficient [22]. Unfortunately, subsequent study demonstrated that the exponential power coefficient between HRV metrics and HR was age dependent and sensitive to the condition/situation in which HRV measured [9]. Until now, none of SCD risk stratification related studies have considered the confounding effect of HR on HRV analysis.

In the present study, we confirmed that most of investigated HRV metrics have an exponential decay relationship between HRV and HR, whether analyzed using long-term or short-term HRV analysis methods. This is in line with Monfredi’s report on the physiological origin of correlation between HRV metrics and HR, although different kinds of subjects were investigated [22]. More importantly, our results demonstrated that the decaying rates were differed among study groups and among HRV metrics. This indicated that the reflex control intensity of autonomic nervous system on heart rhythm was related with the risk degree of SCD. An individualized HR-based HRV adjustment approach was thus proposed and evaluated according to these findings. The experimental results were consistent with the traditional unadjusted HRV analysis methods, that is, HCM patients have autonomic dysfunction, with lower vagal activities and with altered sympatho-vagal balance compared with that of the CON group. However, the differences and trends among groups might be altered for each metric when different methods were applied. For instance, RMSSD and SDNN showed a downward trend if the risk of SCD was medium but the values rises sharply to a far greater extent than the CON group if the risk was high when analyzed using HRV_I_. This phenomenon was only observed in RMSSD when analyzed using HRV_L_ but not shown in HRV_S_. The results of HRV_I_ might reveal the electrical instability of SCD victims with malignant ventricular arrhythmias. Indeed, Huikuri et al. proved that an extremely lower HRV metric indicated true autonomic dysfunction, but a higher value of the metric might not always reflected more healthy autonomic function, it might be an indicator of an unhealthy and highly irregular pattern, such as erratic rhythms [9]. Shaffer et al. also demonstrated that pathological conditions and cardiac conduction abnormalities might increase HRV metrics, and the increased HRV metrics were strongly associated with the increased risk of mortality [26]. Our results also confirmed that the methodologies used have a great impact on HRV analysis and can be used to explain the discrepancies of the clinical studies. More importantly, the ability to stratify the risk of SCD was greatly improved when the proposed method was applied. Such as the AUC values of HRVTI, SDNN and RMSSD were increased from 0.56, 0.58 and 0.59 to 0.73, 0.84 and 0.91, the CV values of HRVTI, SDNN and RMSSD were decreased from 0.21, 0.22 and 0.24 to 0.10, 0.09 and 0.11 respectively when the proposed method was applied. Therefore, our findings have significant implications for the studies that have been and will continue to be carried out on the SCD risk classification, which either did not consider the confounding effect of HR or improperly corrected for the impact of HR when calculating HRV metrics.

This study has several limitations. First, due to the sudden nature of SCD, the sample size of the SCD group was smaller than that of the other two groups. In addition, the ECG duration of this group was also shorter because long-time ECG recording before SCD is very difficult to collect. Second, the clinical spectrum of intermediate risk population is very broad and choice of only HCM patients as the intermediate risk group was an arbitrary decision. Additionally, the clinical features of the HCM patients were not investigated and no follow-up was performed for the participants. Therefore, the incidences of SCD in the normal healthy and in the HCM patients were unknown. At the same time, the data of SCD group were come from public databases and the basic information of these cases was limited. Third, the HRV parameters were demonstrated to be influenced by cardioactive therapies either individually or interconnectedly, but the impacts of these factors on variation in HRV metrics have not been considered. Furthermore, whether the HR-adjusted HRV metrics limit the impact of medical therapy on outcomes remains to be uncertain. Fourth, in order to obtain optimal fitting curve and exponential power coefficient, the proposed method in current study may only applicable to cases with long-term recording together with large fluctuation of HR. But a default fixed coefficient can be used to adjust HRV metrics when only short-term recording is available, although the performance may not as better as using the individualized coefficient.

## Conclusions

HCM patients with medium risk of SCD had similar exponential decay relationship between HRV metrics and HR, but with different decaying rates compared with those of the low risk healthy controls and high risk SCD victims. HCM patients were accompanied by autonomic nervous dysfunction, represented as decreased vagal activity and impaired sympatho-vagal balance when analyzed using the individualized HR-based HRV adjustment method. The HR adjusted HRV metrics provide reliable and robust risk stratification of SCD.

## Data Availability

The datasets used and/or analyzed during the study are available from the corresponding author on reasonable request.
